# Construction and Validation of the modified visual index of parietal atrophy by brain magnetic resonance imaging in Alzheimer's Disease in a memory clinic

**DOI:** 10.1002/alz.088388

**Published:** 2025-01-09

**Authors:** Sara Gloria Aguilar‐Navarro, Daniel Herrera Martínez, Jhonatan Ruvalcaba Ortega, Michelle Gonzalez Putoy, Ivonne Uscanga Mejía, Irvin Abharca Jiménez, Alberto Jose Mimenza

**Affiliations:** ^1^ Instituto Nacional de Ciencias Médicas y Nutrición Salvador Zubirán, México, DF Mexico; ^2^ Instituto Nacional de Ciencias Médicas y Nutrición Salvador Zubirán, Mexico, DF Mexico; ^3^ Instituto Nacional de Cincias Médicas y Nutrición salvador Zubirán, Mexico, DF Mexico; ^4^ Instituto nacional de Ciencias Médicas y Nutrición Salvador Zubirán, Mexico, DF Mexico

## Abstract

**Background:**

Magnetic resonance imaging (MRI) has been very useful in evaluating brain atrophy related to Alzheimer's Disease (AD), so it is important to have scales that allow objective evaluation specifically of parietal atrophy. The objective of this study was to develop and validate a modified visual scale for the assessment of parietal atrophy (APM Scale) by brain MRI in a single slice (axial T2‐FLAIR), with focus on the measurement of the posterior cingulate sulcus to differentiate between Mild Cognitive Impairment (MCI), AD and other forms of dementia (OD not AD).

**Method:**

retrospective validation study carried out in a memory clinic of a tertiary hospital in Mexico City, in a period from March 2015 to June 2023. Patients with a complete clinical and cognitive evaluation were included, including: MMSE, MoCA, brain MRI, Comprehensive Geriatric Evaluation, who met the diagnostic criteria for AD and other dementias and who, according to the evaluation carried out by the group of specialists, were classified into the following groups: Cognitively Healthy (CS), MCI, AD and OD non‐AD. A descriptive statistical analysis to characterize the population, normality tests, ANOVA for continuous variables and chi‐square tests for categorical variables. To demonstrate interobserver agreement, Cohen's Kappa test and the construction of ROC curves were used to determine the sensitivity and specificity of the APM Scale, as well as a logistic regression model to search for its association according to the Gold standard for AD.

**Results:**

400 files were reviewed, 238 were excluded due to lack of data. The average age was 77 SD: 3.2 years, 55.6% were women. Diagnoses by groups included: 12.9% CS, 22.6% MCI, 50% AD dementia and 15.4% other non‐AD dementias. The interobserver agreement for the APM Scale was 0.880 for observer 1 and observer 2, 0.73 for observer 1 and observer 3 and 0.60 for observer 1 and observer 4 (p < 0.001). The AUC for EA was 0.748, with a sensitivity of 72% and a specificity of 71%. (p< 0.001).

**Conclusion:**

This study demonstrates that the APM Scale based on MRI in axial T2 FLAIR sequence is a useful and valid tool to support the diagnosis of AD.

